# Kitchen Girl

**Published:** 2006-09-15

**Authors:** Lynne S. Wilcox

The Appalachian mountains comprise a system of ranges that begins in Quebec, Canada, passes through New York, and reaches south along the U.S. eastern seaboard to Georgia and Alabama. Portions of the mountain system are in 12 states and include ranges such as the Allegheny Mountains of Pennsylvania and the Great Smoky Mountains of Tennessee. The land is heavily forested, rocky, and sparsely populated. Traditionally, the region known as Appalachia offered two sources of income: mining in the north and farming in the south.

In the 18th century, the primary inhabitants of Appalachia were American Indians, particularly the Shawnee tribes in the north and the Cherokee tribes in the south. European immigrants from Scotland, England, and Ireland followed. The descendents of these groups and of southern African Americans now comprise the primary populations of Appalachia. The northern and southern communities of this region have distinct cultures, especially in music. Coal miner songs such as "Sixteen Tons" speak to the dangers and miseries for miners and their families who reside in northern Appalachia. In southern Appalachia, vocal and instrumental music is drawn from the traditional mountain instruments of banjo and fiddle.

But one common cultural trait is shared by northern and southern mountain people: poverty. Overall, the people of Appalachia are unemployed more often than people in the rest of the United States, have a lower median family income, and have lower educational levels (1). They also have higher levels of health-risk behaviors; data from the Behavioral Risk Factor Surveillance System indicate that the region has high levels of smoking and physical inactivity and low rates of mammography and colon cancer screening (1).

Where there is poverty, there are high rates of disease. Compared with the U.S. population as a whole, residents of Appalachia have higher death rates from heart disease, cancer, cerebrovascular disease, obstructive pulmonary disease, diabetes, and infant mortality. In this issue, *Preventing Chronic Disease (PCD)* explores health-risk behaviors among the Appalachian populations. We thank Dr Eugene Lengerich of The Pennsylvania State University for serving as guest editor on this topic.

**Figure:**
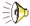
Listen to the instrumental "Kitchen Girl" (MP3–246k) Fiddle Tunes of the Old Frontier: The Henry Reed Collection. Library of Congress, American Folklife Center

Henry Reed (1884–1968) was an accomplished West Virginia fiddler with a broad repertoire of traditional Appalachian tunes. In 1966 and 1967, folklorist Alan Jabbour recorded 184 of Reed's tunes; this collection is now housed in the American Folklife Center at the Library of Congress (2). In the notes accompanying this collection, Jabbour observes that Reed used a variety of musical styles that included bluegrass, ragtime, blues, and popular dance. In particular, instrumental pieces such as "Kitchen Girl" have a melodic line suggestive of eastern woodland American Indian, European immigrant, and African American influences. In an issue devoted to understanding the multiple factors that affect the health of the Appalachian people, it seems appropriate to offer readers of this issue of *PCD* music that reflects the region's multicultural blend.

## References

[1] Haverson J, Ma L, Harner EJ (2004). An analysis of disparities in health status and access to care in the Appalachian region.

[2] Jabbour A Henry Reed: his life, influence, and art. Fiddle tunes of the old frontier: the Henry Reed Collection.

